# Editorial: Innate immunity and severe asthma: From microbiome to target therapy

**DOI:** 10.3389/fimmu.2022.1114275

**Published:** 2022-12-29

**Authors:** Diego Bagnasco, Marco Caminati

**Affiliations:** ^1^ Allergy and Respiratory Diseases, Istituto di ricovero e cura a carattere scientifico (IRCCS) Policlinico San Martino, Genoa, Italy; ^2^ Department of internal medicine (DIMI), University of Genoa, Genoa, Italy; ^3^ Asthma, Allergy and Clinical Immunology Section, Department of Medicine, University of Verona, Verona, Italy

**Keywords:** microbiome, asthma, immunity, T2 inflammation, enterotoxins

An increasing amount of evidence supports the role of microbiome as a major player in the pathobiology of several conditions. Most of the research on the topic has so far investigated gut microbiome, but a number of studies have recently focused on other districts including the respiratory tract. Although it has been considered for a long time a sterile organ without any commensal population, according to the latest evidence the lung microbiome seems to be primarily involved in the immunological background of respiratory tract diseases (1).

The increasing interest in microbiome has been supported by some methodological innovations in the field, in terms of omics sciences, combining the more traditional culture and incubation on media with the newer protein chain reaction (PCR). Relying on those techniques large databases including genomes and metagenomes of the human microbiome have been developed, enabling the investigation of the functional effects of the human microbiome through the detection of functional genes encoded by the microbial community and their products (proteins, metabolites, etc.) (2).

Although the permanent cross talking between the environment and the skin, gut and respiratory tract surfaces exerts an inevitably dynamic effect on microbiome (3), the first two months after birth remain critical for the growth of the commensal microorganisms pool in the respiratory tract (4). The development of the immune tolerance to environmental agents, including allergens, also takes place in the first few weeks of life, and seems to be heavily affected by the microbiome composition (5).

Under that perspective, the relevance of environmental determinants characterizing the early life “exposome” of each individual, including hygienic conditions but also feeding habits, is quite expected.

Breastfeeding, for example, has been associated with a reduced likelihood of developing asthma later in life, compared with infants who were not naturally breastfed (6). It has been described that alterations in the pulmonary micro biota, starting at birth, lead to the development of a variety of chronic respiratory diseases such as asthma (7). Of note, the specific composition of lung and respiratory tract microbiome seems to substantially contribute to different inflammatory patterns and consequently to the definition of the asthma endotype. According to some evidence coming from murine models reproducing chronic lung inflammation the enrichment of pseudomonas and lactobacillus, derived from affected human subjects, stimulates a TH 17-driven response, while pathogens such as proteobacteria induces severe airways inflammation mediated by the activation of a TLR2-independent pathway (4).

The relevance of microbiome has been explored also in lung carcinoma, specifically adenocarcinoma, whose pathobiology seems to be related to the presence of specific commensals facilitating the production of interleukin (IL) - 17 through the stimulation of T γδ cells (8).

Regarding allergic asthma, some authors have postulated a link between the presence and diversity of dust bacterial content and susceptibility to allergic sensitization to mites; however, a clear-cut association between pulmonary bacterial dysbiosis and type 2 (T2) inflammation has not been demonstrated so far (9). Some authors speculated about a major role of fungi more than bacteria in the induction of a T2 response., but there is still little evidence on that (10, 11). A recent study compared the microbiome of two subpopulations, namely patients with mild asthma and atopic subjects without asthma, with a control group of healthy nonatopic patients; the authors observed a specific pattern of dysbiosis strongly which was strongly associated with atopy alone, and a different one associated with asthma (12). Although the results showed that a particular microbiome might favor one type of disease over another, the differentiating factors are not known in details. On the opposite, in the case of neutrophilic asthma, an altered composition of the bacterial microbiota has been observed to be present, when compared with T2 endotype (13). Haemophilus, Klebsiella, Moraxella, and Pseudomonas, represent the most frequently detected bacteria, although in several studies (1, 14, 15) severe asthma patients with neutrophilic inflammation showed a wide diversity of the microorganisms composing the airway bacterial flora, compared with a more limited variability characterizing eosinophilic or pauci-granulocytic patients. Other studies have also confirmed a direct relationship between the abundance of microorganisms from the proteobacterial family and IL-17 (13), as well as a correlation between the above-described bacteria with increased cytokine levels and worse disease control. Under that perspective, the role of staphylococcus aureus and its enterotoxins (SEs) in inducing T2 inflammation in subjects with severe asthma, recently confirmed and further investigated by studies in Real Life (16, 17) another example of the cross-talking between microorganisms and the immunological background underlying bronchial inflammation.

Although not yet fully understood, the relationship between commensal and non-commensal microorganisms and the immune system and its relevance in the development of asthma represents a new perspective to look at asthma pathobiology (**Figure 1**). At the same time it paves the way to potential primary prevention strategies, in terms of early life or interventions, as well as to innovative targeted therapeutic approaches in affected individuals, especially those suffering from difficult to treat asthma phenotyopes. It implies further development of dedicated tools, such as omics sciences, in terms of translational research and their implementation in clinical practice.

**Figure 1:**
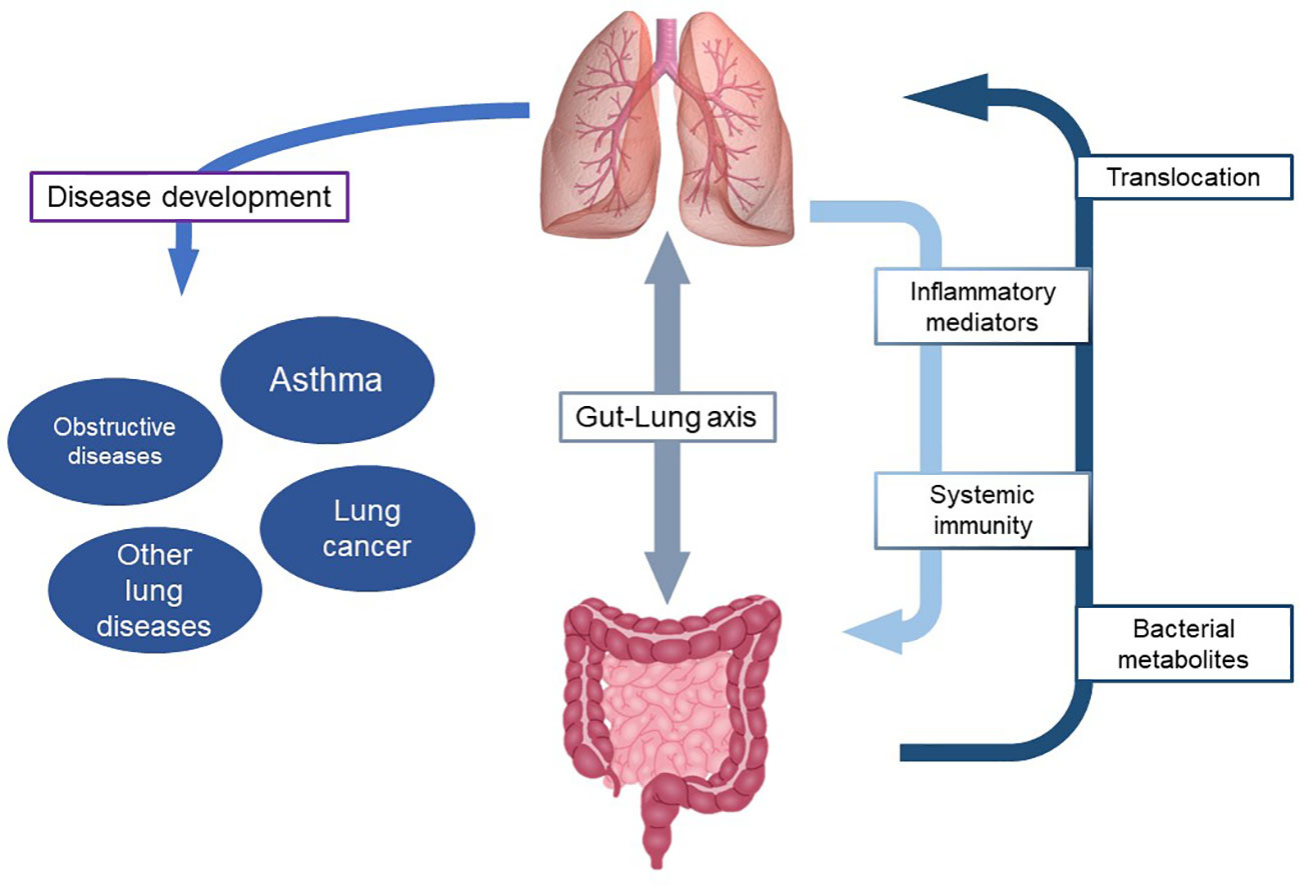
Role of microbiome in the interaction between gut and lung and development of respiratory diseases.

## Author contributions

DB and MC equally contribute to the development and writing of manuscript. All authors contributed to the article and approved the submitted version.
